# Hyperspectral Image-Based Variety Classification of Waxy Maize Seeds by the t-SNE Model and Procrustes Analysis

**DOI:** 10.3390/s18124391

**Published:** 2018-12-11

**Authors:** Aimin Miao, Jiajun Zhuang, Yu Tang, Yong He, Xuan Chu, Shaoming Luo

**Affiliations:** 1 College of Automation, Zhongkai University of Agriculture and Engineering, Guangzhou 510225, China; am_miao@zju.edu.cn (A.M.); zhuangjiajun@zhku.edu.cn (J.Z.); chx0105@cau.edu.cn (X.C.); smluo@gdut.edu.cn (S.L.); 2 College of Biosystems Engineering and Food Science, Zhejiang University, Hangzhou 310058, China; yhe@zju.edu.cn

**Keywords:** hyperspectral imaging, waxy maize, variety classification, t-SNE, procrustes analysis

## Abstract

Variety classification is an important step in seed quality testing. This study introduces t-distributed stochastic neighbourhood embedding (t-SNE), a manifold learning algorithm, into the field of hyperspectral imaging (HSI) and proposes a method for classifying seed varieties. Images of 800 maize kernels of eight varieties (100 kernels per variety, 50 kernels for each side of the seed) were imaged in the visible- near infrared (386.7–1016.7 nm) wavelength range. The images were pre-processed by Procrustes analysis (PA) to improve the classification accuracy, and then these data were reduced to low-dimensional space using t-SNE. Finally, Fisher’s discriminant analysis (FDA) was used for classification of the low-dimensional data. To compare the effect of t-SNE, principal component analysis (PCA), kernel principal component analysis (KPCA) and locally linear embedding (LLE) were used as comparative methods in this study, and the results demonstrated that the t-SNE model with PA pre-processing has obtained better classification results. The highest classification accuracy of the t-SNE model was up to 97.5%, which was much more satisfactory than the results of the other models (up to 75% for PCA, 85% for KPCA, 76.25% for LLE). The overall results indicated that the t-SNE model with PA pre-processing can be used for variety classification of waxy maize seeds and be considered as a new method for hyperspectral image analysis.

## 1. Introduction

Waxy maize (also called waxy corn or glutinous maize) contains a large amount of amylopectin and is favoured by consumers around the world, especially in China [1,2]. Variety discrimination is an important step in maize seed quality testing that can prevent the use of false and inferior seeds and protect intellectual property rights [1].

Hyperspectral imaging (HSI) is an emerging technique that combines machine vision and spectroscopy systems and is becoming widely used in the variety classification of wheat [3,4], rice [5,6], cotton [7], and grape [8] seeds due to its decreased time requirements and material consumption benefits compared with traditional morphology or molecular identification methods [9,10]. In addition, the application of HSI in the variety classification of maize seed kernels also exhibits good performance [1,11,12,13,14], and even the near-infrared bands of the spectral imaging technique can be used to achieve good variety classification results of coated maize seeds [15]. However, there are few studies that classify waxy maize seeds using HIS technology.

First, due to improved sensor technology, compact data formats and rapidly increasing digitalization capabilities, the amount of spectral data has increased dramatically in the past decade. As a consequence, dimensionality reduction (DR) has become a key tool in spectra analysis. Most traditional studies are based on the linear assumption of hyperspectral images, and classical linear DR methods, such as PCA (principal component analysis) and ICA (independent component analysis), are widely used in HSI studies [16,17]. However, due to intra-pixel spectral mixing, scene heterogeneity and the complex processing of HSI, the original spectral features are nonlinearly correlated. It is desirable to explore in-depth nonlinear data relationships, though very limited work related to this problem has been conducted. 

Nonlinear data processing is a challenging problem that has drawn increasing attention. Available solutions can be divided into two categories: kernel-based and manifold learning methods. The kernel-based methods (such as kernel principal component analysis, KPCA) have been proven to be effective for non-linear data analysis, while the kernel function plays a critical role in KPCA; however, there are no rules for choosing the function, and the performance will be significantly impaired by a poor choice [18]. Currently, the study of DR approaches focuses on the manifold learning methods, and the t-distributed stochastic neighbourhood embedding (t-SNE) is becoming a key focus of them. t-SNE is a local probability-based non-linear DR algorithm; it is a variation of stochastic neighbour embedding (SNE) via which it is much easier to produce significant visualizations by reducing the tendency to crowd points together in the centre of the map. For example, t-SNE can reveal natural classes of the MNIST (a standard benchmark for classification and computer vision systems) without supervised information, whereas other conventional algorithms, such as isometric feature mapping (Isomap), locally linear embedding (LLE), and Sammon mapping, fail [19]. Thus, t-SNE has received increasing attention and has been successfully applied to the analysis of bird songs [20], computational fluid dynamics [21], genomic data [22], remote sensing images [23] and so on.

Second, most existing image analysis methods assume that the training set and the test set are in exactly the same experimental conditions and data distribution, though this assumption is invalid in most cases due to the light or position variations under even the same conditions. Thus, the shifting of figures must be considered, and data adjustment should be previously managed for the actual process. To solve this problem, data smoothing, data denoising and first derivation approaches were used, though these approaches were limited because they only consider one aspect of the data [24]. Procrustes analysis (PA) is a method that focuses on this problem by matching the data variable correlations from one set to another. By minimizing a measure of shape difference optimally through successive iterations, PA can efficiently eliminate the influence of the shifting of data. PA has been successfully used for statistical shape analysis and image alignment [25,26]. Based on its competitive advantage, PA was used in this study as a preprocessing method in the hyperspectral image processing to eliminate the translation, rotation and scaling components caused by different angles of spectral sensors, lamps and imaged samples, etc. 

Finally, the widely used method of Fisher’s discriminant analysis (FDA) was applied for waxy maize classification. After the local discriminant information was obtained by t-SNE, the global discriminant information was achieved as a complement by maximizing the between-class scatter and minimizing the within-class scatter in FDA [27,28,29].

The purpose of this study was to investigate the feasibility of using PA and t-SNE for the classification of waxy maize seed varieties based on hyperspectral imaging. The specific objectives were to (1) compare the performance of the t-SNE-, PCA-, KPCA- and LLE-based classification models; and (2) improve the classification accuracy by data preprocessing using the PA algorithm; and (3) compare the differences of hyperspectral images between the embryo side and non-embryo side of maize seeds. 

## 2. Materials and Methods

### 2.1. Samples

**Figure 1:**
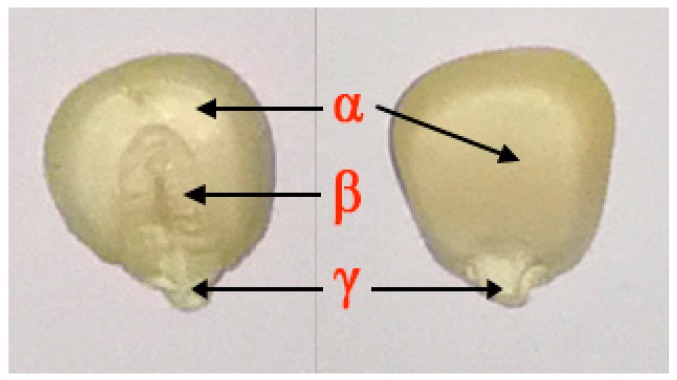
The structure of waxy maize seeds. The left shows the embryo side, and the right shows the non-embryo side, where (α) Endosperm, (β) Embryo, (γ) Tip cap (fruit stalk).

**Figure 2:**
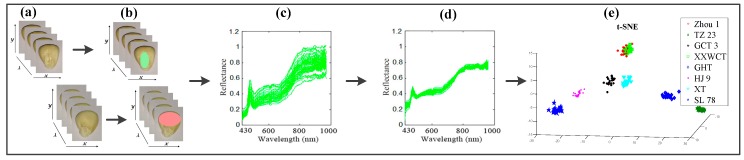
Flowchart of the image processing and data analysis for variety classification of waxy maize seeds. (**a**) the experimental seeds; (**b**) region of interest (ROI) selection from the seeds using ENVI software; (**c**) spectral data extraction and calibration; (**d**) spectral data preprocessing using Procrustes analysis (PA) algorithm; (**e**) seed variety classification by t-distributed stochastic neighbourhood embedding (t-SNE) and other models using MATLAB software.

Seeds of eight waxy maize varieties (Zhou 1, TZ 23, GCT 3, XXWCT, GHT, HJ 9, XT and SL78) were studied; they represented the common varieties grown in China and were purchased from a seed company (FMYS Technology Ltd., Beijing, China). The seeds were sealed in plastic bags and stored in a refrigerator at 4 °C until the HSI process, and their moisture content was 7% to 8%. Considering the imparity of maize seeds, both the embryo and non-embryo sides of every seed were explored; in this study, a total of 800 maize kernels (100 kernels per variety, of which 50 seeds were imaged on the embryo-side and the other 50 seeds on the non-embryo side) were randomly selected, all of which were similar in size and structurally intact (see Figure 1 and Figure 2a). The samples of each variety were divided into a train set and a test set by Kennard-Stone algorithm in a ratio of 4:1. Therefore, for each variety, there were 40 samples in the training set and 10 samples in test set per side of the seed. In the following step, the model construction and testing were implemented by the train set and test set, respectively. 

### 2.2. Hyperspectral Imaging System

The visible/near-infrared hyperspectral imaging system (GaiaSky-mini, Dualix instruments Ltd., Sichuan, China), with 256 bands in the spectral ranges of 386.7–1016.7 nm, includes a CCD camera (ICX285, Sony, Tokyo, Japan) with a spectrum resolution of approximately 3 ± 0.5 nm, two 50-W LED lamps for illumination, and a conveyer belt driven by a stepping motor (ZOLIX SC300, Zolix instruments Ltd., Beijing, China). This system is controlled by a computer with SpecView software (Dualix instruments Ltd., Sichuan, China).

### 2.3. Spectral Data Extraction and Calibration

Both sides of each waxy maize seed, the embryo (left seed in Figure 2a) and non-embryo (right seed in Figure 2a), were placed on the white stage in the same arrangement (8 rows × 8 columns) and then transferred to the camera to be scanned line by line at 1.2 mm/s with a 15-ms exposure time to acquire three-dimensional (*x*, *y*, *λ*) hyperspectral images; the images were acquired with 512 pixels in the *x* direction, 672 pixels in the *y* direction, and 386.7–1016.7 wavelengths in the *λ*-direction (Figure 2a).

After the acquisition of hyperspectral images, a series of steps were carried out to extract and calibrate the spectral data. First, the beginning and ending ranges were omitted from each spectral data, as they were heavily influenced by stochastic noise [11]; therefore, 220 spectral bands from 430.1 nm to 971.5 nm were selected for further analysis. Second, the embryo part was segmented from the embryo side of the seed by an ellipse shape to create the region of interest (ROI), and the endosperm part was segmented from the endosperm side of the seed by an ellipse shape to create the ROI. Then, the spectral data within the ROIs (ellipse shapes) were extracted and averaged at each wavelength by using ENVI 5.1 (see Figure 2b) to create a mask of the region of interest (ROI), and then the spectral data within the ROI were extracted and averaged at each wavelength by using ENVI 5.1 (ITT Visual Information Solutions, Boulder, CO, USA). 

Finally, to calculate the reflective spectrum, the spectral raw image (*I*_raw_) of the maize seeds was calibrated using two reference criteria: the “white” image (*I*_white_) was obtained using a standard white Teflon tile that is configured with the machine to establish the maximum reflective conditions, and the “dark” image (*I*_dark_) was acquired by turning off all the light sources and covering the lens with its original cap to define the non-reflective condition. The calibrated image (*I*) was eventually acquired using formula (1) to correct the effect of light intensity on the images [30] (see Figure 2c):

(1) $$I=\frac{I_{raw}-I_{dark}}{I_{white}-I_{dark}}$$

### 2.4. Optimal Wavelength Selection

Each extracted spectrum consisted of 220 spectral bands ranging from 430.1 to 971.5 nm. As a small number of variables can reduce the redundancy and computation, it was expected that fewer bands represent most of the useful information. The successive projection algorithm (SPA) is a forward optimal waveband selection method that can minimize the collinearity among variables by simple calculations and has been successfully applied in previous studies [11,31]. In this study, SPA was used to test the optimal wavelengths, and the root mean square error (RMSE) in cross-validation was used to evaluate the model performance [31].

### 2.5. Spectra Data Preprocessing and Analysis

As the condition change may have great effect on the image data, the PA was used to remove the translation, rotation and scaling components of the hyperspectral data. For the data **Y**, to best match the shape of the target points **X** in the same class, the optimal transformation was defined in PA as the smallest sum of the squared distances among **X** and **Y**. The optimal alignment of **X** and **Y** was achieved by minimizing the following Equation:

(2) $$E={\left\|X-Y\right\|}_{F}^{2}=\mathrm{trace}(X-Y)(X-Y{)}^{T}=\sum_{i=1}^{N}(x_{i}-y_{i}){\left(x_{i}-y_{i}\right)}^{T}$$

where ${\left\|\cdot \right\|}_{F}$ was the Frobenius norm. The above problem is solved by the translation vector **T**, rotation **R** and scale factor **s**. By defining ${\hat{X}}_{i}=sX_{i}R_{i}+T_{i}$, the data matching deviation was measured by minimizing the following objective functions [25]:

(3) $$E(s,R,T)={\left\|X-Y\right\|}_{F}^{2}={\left\|sXR^{T}+T-Y\right\|}_{F}^{2}$$

After reducing the spectral data to a low-dimensional space by t-SNE (Figure 2e), the Euclidean distances between data points in high-dimensional space were converted into conditional probabilities. The similarity of data point **x***_j_* to data point **x***_i_* was represented by the conditional probability *P_j|i_*. **x***_i_* would pick **x***_j_* as its neighbour if neighbours were picked in proportion to their probability density under a Gaussian centred at **x***_i_*. For nearby data points, *P_j|i_* was relatively high, whereas for widely separated data points, *P_j|i_* will be almost infinitesimal (for reasonable values of the variance of the Gaussian, *σ_i_*). Mathematically, the conditional probability *P_j|i_* was given as:

(4) $$P_{j\;|i+k}=\frac{\exp(-\left\|x_{i}-x_{i+k}\right\|/2\sigma_{{}_{i}}^{2})}{\sum_{k\neq 0}\exp(-\left\|x_{i}-x_{i+k}\right\|/2\sigma_{{}_{i}}^{2})}$$

where *σ_i_* was the variance of the Gaussian that is centred on data point **x***_i_*. To solve the out-of-sample problem, the parametric t-SNE technique [32] was used in our study to learn a parametric mapping between the high-dimensional data space and the low-dimensional latent space for the test data. For more details of t-SNE, see the reference section [19,32]. 

In this work, FDA was used for classification and recognition of the low-dimensional image data. The performance of the classification model was verified by classification accuracy, which was defined by the ratio of the samples correctly classified to the total samples.

### 2.6. The Training, Testing and Validation of the Model

**Figure 3:**
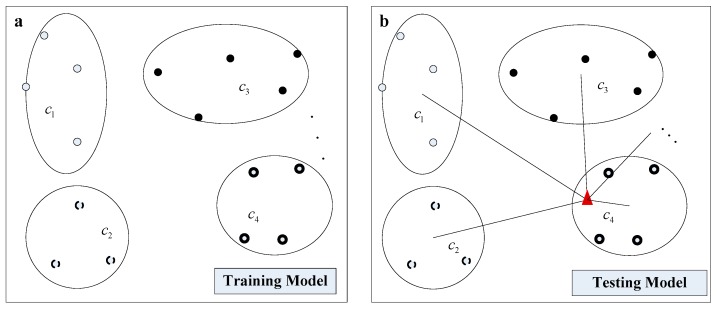
Two procedures for the variety classification (**a**) The training of the model by the labelled samples, (**b**) variety reorganization for the test data. The four ellipses represent four varieties, the other four varieties are omitted in the figure. The red point in Figure 3b represents the seeds of one of the eight varieties, which was prepared for reorganization.

In the proposed method, a classification model was initially built by the train set which includes the spectral data of all the eight varieties. Based on the proposed model, the maize varieties for a separate new unlabelled test set were recognised. The procedure of the training and testing model were illustrated in Figure 3. The variety of the test seed (red point in Figure 3b) was determined as the one with the nearest distance among the spectral data of eight varieties. 

**Figure 4:**
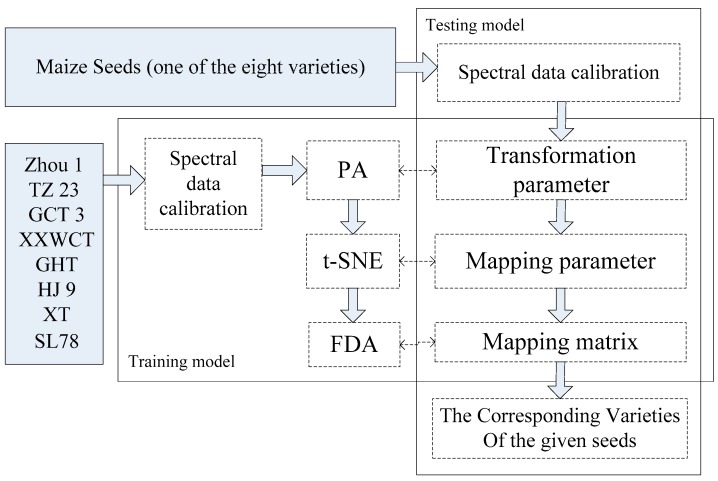
The detailed procedure of the training and testing model for variety classification of waxy maize seeds.

In the model construction procedure, the optimal model parameters for PA, t-SNE, FDA that can optimally discriminate different varieties of the seeds were identified after data calibration. Based on the constructed model and the model parameters, seed variety of the unlabelled test set which were not included in the train set can be predicted in the testing procedure. It was noticed that the seed variety was determined as one of the eight varieties, regardless of whether they actually belong to the scope of eight varieties or not. The model performance was evaluated by the classification accuracy of the test set. The detailed procedure was illustrated in Figure 4.

In this study, the model validation was also carried out by hold-out cross-validation. The train set applied above were randomly divided into 5 separate subsets and the validation was implemented by choosing one subset as the validation data iteratively until all of the divided datasets were processed. The above model training and validation were conducted for 10 times by forming the training data for different subsets. All images were analysed using MATLAB version R2014a (The Math Works, Natick, MA, USA). To compare the effect of t-SNE-, PCA-, KPCA- and LLE-based classification models with or without PA preprocessing, a total of eight comparative combinations (PCA+FDA, KPCA+FDA, LLE+FDA, t-SNE+FDA, PA+PCA+FDA, PA+KPCA+FDA, PA+LLE+FDA, PA+t-SNE+FDA) were established in this study.

## 3. Results and Discussion

### 3.1. Optimal Spectral Wavebands

**Figure 5:**
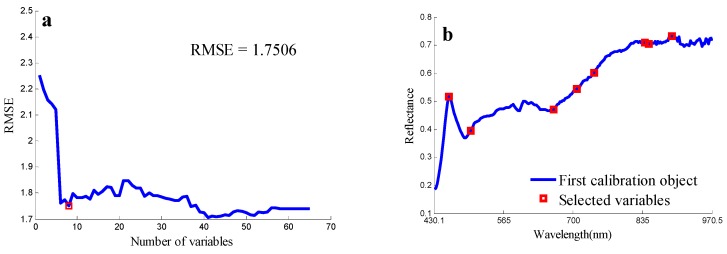
The RMSE plot (**a**) and selected wavelengths (shown in red square marker) by SPA (**b**). RMSE: root mean square error. SPA: successive projection algorithm.

The optimal wavelengths selected for the non-embryo side by SPA was shown in Figure 5. The RMSE value was significantly decreased when the number increased from 1 to 8 in Figure 5a. The optimal eight spectral wavebands were shown in Figure 5b (455.5 nm, 697.6 nm, 495.1 nm, 841.2 nm, 732.4 nm, 653.3 nm, 887.7 nm, 833.6 nm), which indicates that they had the greatest ability to discriminate the samples and they could be used to develop the propose classification model. The optimal spectral wavebands of empro images were also selected by the same method, which is very different to the non-empro side as the empro side contains starch, oil (in the embryo), and other chemical compounds. The most optimal Spectral Wavebands were selected as 737.4 nm, 578.1 nm, 460.2 nm, 932 nm, 937.2 nm, 945.1 nm, 947.7 nm and 960.9 nm.

### 3.2. Spectral Features of Waxy Maize Seeds

**Figure 6:**
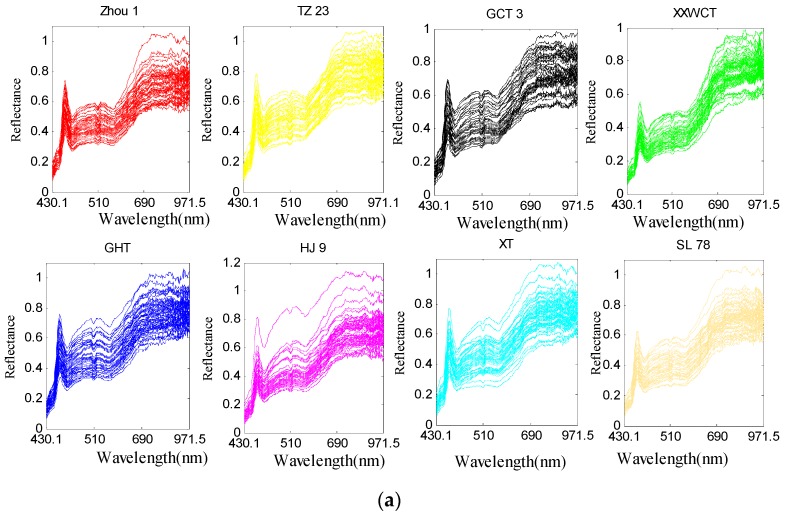

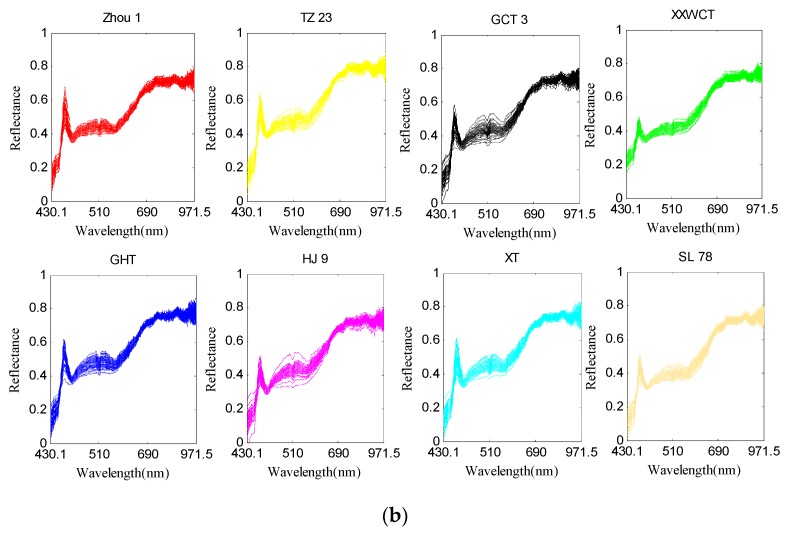
The processing of Procrustes Analysis (PA): (**a**) Raw spectra of eight maize varieties in the train set, and (**b**) PA-preprocessed data for the raw spectra. The abscissa axis represents the unit of the wavelength (nm), and the ordinate axis shows the relative reflectance.

**Figure 7:**
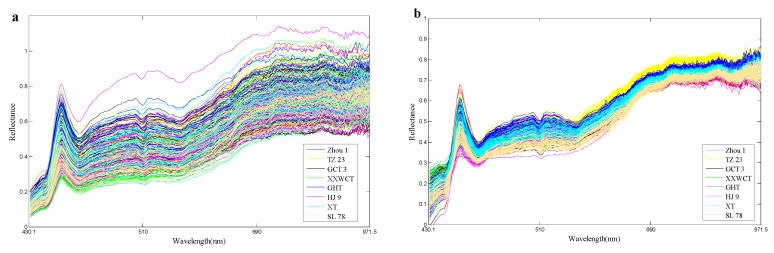
(**a**) Raw spectra of eight maize varieties in the train set, and (**b**) PA-preprocessed data for the raw spectra.

Although the hyperspectral images were corrected before analysis, noise still existed. To avoid significant noises, spectra from 430.1 to 971.5 (bands 20 to 239) were used for analysis. Raw spectral profiles of the eight waxy maize seed varieties were shown in Figure 6a; for the same maize variety, all the hyperspectral images were widely scattered during their spectra along the bands as the light and noise affected. In such case, the data in different classes may be overlapped, and the classification model may also be influenced eventually. The hyperspectral images with the same seed variety were likely to be distributed with the same variation and minimized deviation. The PA algorithm aimed to decrease the data variance in such a manner so as to minimize the differences in the hyperspectral data. With such excellent property, the PA algorithm was appropriate to be utilized in preprocessing the raw spectra. The obtained pretreatment spectral curves were shown in Figure 6b. It can be seen that the PA preprocessed spectra agglomerate and strengthen the information of the raw spectra. A comparison of the eight maize varieties for the train set was given in Figure 7. PA in this study was used as a preprocessing method to align the hyperspectral data of the train set. It is noticed from Figure 7 that the strongly overlapping data in Figure 7a were clearly missing after the PA preprocessing, which made a significant contribution to the variety identification of maize seeds.

### 3.3. Multivariate Data Analysis

**Figure 8:**
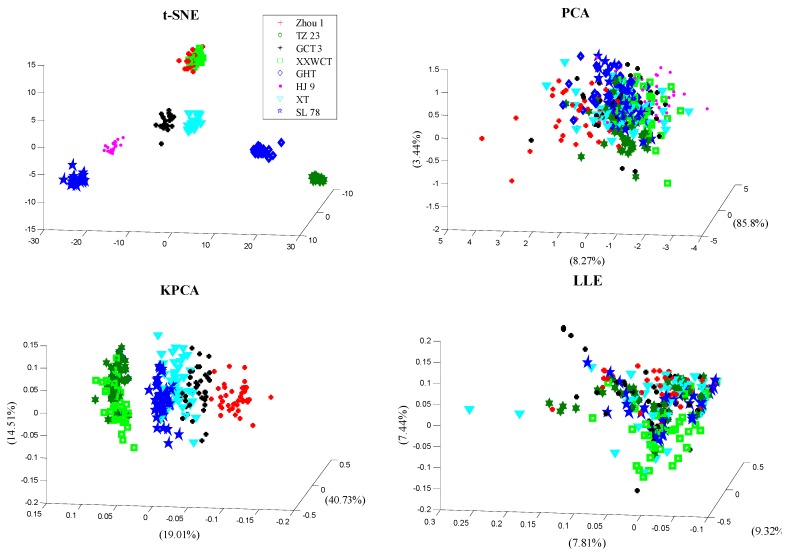
Scores scatter plots of samples from eight maize varieties, using the methods of t-SNE, PCA, KPCA and LLE.

In this section, the performance of the proposed PA and t-SNE based classification strategy was illustrated through eight maize varieties, and the traditional widely used dimensionality reduction methods PCA, KPCA and LLE were also implemented in comparison. The Gaussian kernel k(x,y) = exp(−‖x − y‖^2^/σ) was used as the kernel function in KPCA, where the kernel parameter σ was set as 50. The number of nearest neighbours was set to 8 in LLE. The three-dimensional score plot for the eight maize varieties is shown in Figure 8. For PCA, no significant separation between the different varieties was observed in Figure 5, and the samples in the same class were distributed and overlapped along the space. The scatter plot of LLE was almost the same as that of PCA. The distinction effect of KPCA was better than that of PCA and LLE but worse than that of t-SNE; there was a clear distribution among the Zhou 1, TZ 23, XXWCT, GHT, but the scatter was crowded and even overlapped. The t-SNE showed excellent performance, and there was a clear distribution and area of distinction among the variety TZ 23, GCT 3, GHT, HJ 9, XT and SL 78, while the Zhou 1 and XXWCT were in the same distribution area and cross to a certain extent. Clearly, the varieties in the score plot were more separate by the t-SNE method than the other methods. This was consistent with the previous publications, which found that the performance of t-SNE was better than other non-linear methods in data clustering [20,21,22,23]. That was due to the advantages of t-SNE in capturing local data characteristics and subtle data discriminate structures; the data were departed as shown in the original publication [19]. The waxy maize seeds with the similar geometric structure always gave absolutely similar hyperspectral images, and the adjacent spectral curves in the “Reflectance-wavelength” figures exhibited high dependency. Thus, using the local feature of the spectral curves was usually more reliable in exploiting the underlying discrimination information because the detailed data variations can be easily expressed, and such property showed a competitive advantage for the variety classification. For PCA and KPCA, the only focus was on explaining most of the data variance, and the data distinction failed to be exhibited. LLE mainly focused on preserving the local geometry variation after the nonlinear mapping. As the faraway points were not defined in LLE, the adjacent points may be mapped deceptively close, even resulting in overlapping in the low-dimensional space, as shown in Figure 8. 

After the PA preprocessing and DR process by t-SNE, PCA, KPCA and LLE, the FDA algorithm was finally used to illustrate the performance for variety classification of maize seeds. Table 1 summarises the detailed results based on these models. were chosen based on the minimum root-mean-square error of cross validation.

For models using seed embryo-side data, the accuracies of the PCA+FDA, KPCA+FDA, LLE+FDA and t-SNE+FDA models were nearly 50%, while the accuracies increased to 62.5~87.5% by employing PA. For models using non-embryo-side data, all of the accuracies surpassed 60%. Interestingly, the eight models using the non-embryo-side data were more accurate than the embryo side.

For models without PA, the test accuracies were only 35~71.25%. In contrast, the models with PA showed better results with an accuracy of 62.5~97.5%. The significant effect of PA may be due to the fact that it is an agglomerate effect, which has been shown to be useful for classification in different fields [25,26]. Without PA preprocessing, KPCA showed similar classification accuracy to t-SNE, especially for the embryo-side data, but the accuracy was worse than that of t-SNE with PA preprocessing. This result was consistent with previous reports [18], which showed the instability and the importance of kernel function selection in KPCA. As a kernel-based algorithm, different choices had great influence on the performance of KPCA, and it was hard to give a stable and robust classification result. 

LLE also exhibited poor performance; although the algorithm focused on the local structure of the data, it was difficult to reflect the differences between the distant samples, and the data will congest after the DR process [19]. However, the data in low-dimensional space of t-SNE were regarded as a t-distribution, and the distance between different clusters was enlarged, which was a huge advantage compared with LLE and other manifold learning methods [20,21,22,23].

Obviously, the accuracy of the t-SNE model was higher than that of other models, and the t-SNE model with PA preprocessing exhibited the best result among all methods, with an accuracy of 97.5% using the non-embryo-side data. The best accuracy of other models only reached 85%. In general, except for the KPCA+FDA model, the t-SNE model performed better than other models using each sides’ data with or without PA preprocessing. 

The accuracy obtained by 10 times hold-out cross-validation was 0.85 ± 0.05, demonstrating that the classifier trained using the presented method was robust for the variety classification, and furthermore, it also indicated that the prediction result using the test dataset would be reliable.

## 4. Conclusions

The variety of waxy maize seeds was classified from hyperspectral data based on t-SNE, PCA, KPCA and LLE models. The main conclusions were as follows:

(1) The accuracy of the t-SNE model was higher than that of the PCA, KPCA and LLE models without PA preprocessing, and the classification accuracy of the t-SNE model was 71.25%, which was much more satisfactory than the results of PCA, KPCA and LLE models (with accuracies of 35~65%).
(2) The accuracy of the t-SNE, PCA, KPCA and LLE models was improved by PA preprocessing for the variety classification of maize seeds, and the best classification result, with an accuracy of 97.5%, was obtained by the t-SNE model with PA preprocessing.
(3) All of the models using the non-embryo-side data were more accurate than the embryo-side in the variety classification.

The above conclusions showed that the HSI technology could be improved by using the t-SNE model and PA preprocessing, which can be readily applied to the variety classification of waxy maize seeds. Further studies are expected to conduct more research on how to improve the robustness and universality of these discrimination models by using more varieties or samples, comparing the impacts of different HSI systems, constructing some standard discrimination models and so on. 

## Figures and Tables

**Table 1:** Classification results of the test set for eight waxy maize varieties using spectra data.

Preprocessing	Model *	Embryo Side		Non-Embryo Side	
		Nr/Nt *	Accuracy **	Nr/Nt	Accuracy
None	PCA+FDA	38/80	47.50%	49/80	61.25%
None	KPCA+FDA	42/80	52.50%	52/80	65.00%
None	LLE+FDA	58/80	35.00%	48/80	60.00%
None	t-SNE+FDA	41/80	51.25%	57/80	71.25%
PA	PCA+FDA	60/80	75.00%	60/80	75.00%
PA	KPCA+FDA	50/80	62.50%	68/80	85.00%
PA	LLE+FDA	57/80	71.25%	61/80	76.25%
PA	t-SNE+FDA	70/80	87.50%	78/80	97.50%

* Nr is the number of samples correctly classified; Nt is the total number of samples. ** Accuracy is the classification accuracy of the test set.
